# Localization of Ceramide Synthase 5 in Mouse Retina

**DOI:** 10.1002/cne.70192

**Published:** 2026-07-24

**Authors:** Soo‐Jin Song, Jae‐Hyun Koo, Hee‐Yeon Kim, Joo‐Won Park, Sun‐Sook Paik, In‐Beom Kim, Jung‐A. Shin

**Affiliations:** ^1^ Department of Anatomy Ewha Womans University College of Medicine Seoul South Korea; ^2^ Department of Biochemistry Ewha Womans University College of Medicine Seoul South Korea; ^3^ Department of Anatomy College of Medicine The Catholic University of Korea Seoul South Korea

**Keywords:** ceramide, ceramide synthase 5, electroretinography, retinal function, retinal localization, retinal morphology

## Abstract

Ceramide synthases (CerS) are key enzymes in sphingolipid metabolism that regulate fundamental cellular processes, including apoptosis, cell growth, and homeostasis. Among the six known mammalian isoforms (CerS1–CerS6), CerS5 has been particularly well studied for its involvement in the synthesis of the sphingolipid C16‐ceramide. However, its expression, localization, and functional significance of CerS5 in the retina remain unclear. In the present study, we investigated the presence, distribution, and functional role of CerS5 in mouse retina using CerS5 knockout (KO) mice. We performed quantitative polymerase chain reaction, X‐gal staining, and immunohistochemistry to analyze the expression and localization. Electroretinography (ERG) was employed to assess the impact of CerS5 deficiency on retinal function. Our results demonstrated that CerS5 is localized to the inner nuclear layer and ganglion cell layer, co‐localizing with horizontal cells and specific subsets of amacrine and ganglion cells. The retina of CerS5 KO mice showed a reduction in overall thickness, with significant thinning observed in all retinal layers except the photoreceptor, whereas the outer plexiform layer showed increased thickness. Despite these structural alterations, ERG recordings revealed no significant changes in retinal function. These findings suggest that CerS5 contributes to the maintenance of retinal structural integrity, particularly through its presence in specific retinal cell types, whereas its loss does not markedly impair retinal function in adult mice. The observed structural alterations highlight its potential role in retinal physiology and possible implications for retinal pathophysiology, warranting further investigation into compensatory mechanisms by other ceramide synthase isoforms.

## Graphical abstract

CerS5 localizes to amacrine cells, horizontal cells, and retinal ganglion cells in the mouse retina. Its deficiency reduces C16‐ceramide and causes retinal thinning without functional ERG deficits, suggesting a structural role for CerS5 in retinal integrity.

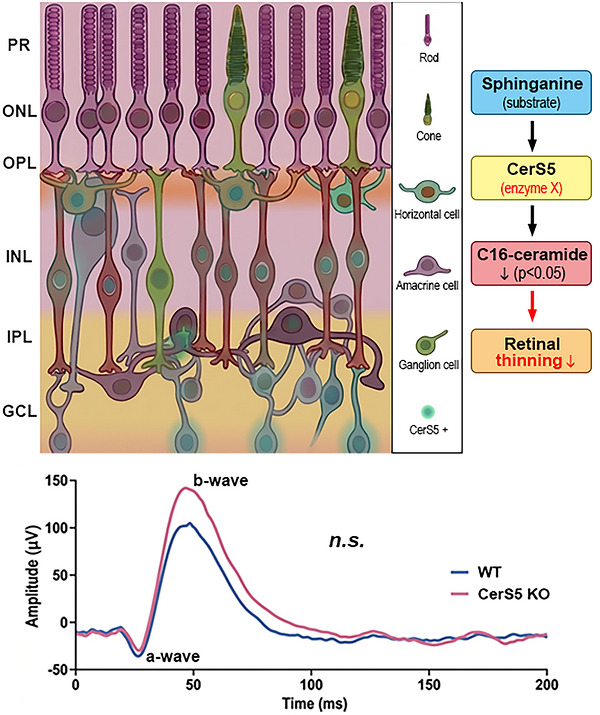

**Abbreviations**
*CerS* and CerS: ceramide synthase
ChAT: choline acetyltransferase
ERG: electroretinography
GABA: γ‐aminobutyric acid
GCL: ganglion cell layer
GS: glutamine synthetase
INL: inner nuclear layer
IPL: inner plexiform layer
KO: knockout
*LacZ* and LacZ: β‐galactosidase
mRNA: messenger RNA
ONL: outer nuclear layer
OP: oscillatory potential
OPL: outer plexiform layer
P0: postnatal day 0
PBS: phosphate‐buffered saline
PKCα: protein kinase C alpha
PR: photoreceptor
RBPMS: RNA‐binding protein with multiple splicing
RGC: retinal ganglion cell
ROI: region of interest
SMI‐32: non‐phosphorylated neurofilament H
TH: tyrosine hydroxylase
WT: wild type

## 1 Introduction

As an integral membrane protein of the endoplasmic reticulum (Stiban et al. 2010), ceramide synthase (CerS) catalyzes the synthesis of ceramide from sphingoid long‐chain bases and fatty acyl‐CoA and plays an important role in regulating sphingolipid metabolism (Gosejacob et al. 2016; Mullen et al. 2012). Ceramide is a bioactive sphingolipid that is involved in the cell cycle, regulating growth, apoptosis, and signal transduction and maintaining cellular homeostasis (Haimovitz‐Friedman et al. 1997; Jayadev et al. 1995; Pettus et al. 2002; Ruvolo 2001, 2003). With its high metabolic demand and complex structure, the retina is particularly influenced by sphingolipid metabolism and ceramide homeostasis, which are critical for the survival and function of retinal cells under oxidative stress (German et al. 2006; Sanvicens and Cotter 2006; Simon et al. 2021).

In mammals, CerS is present in six isoforms (CerS1–CerS6), each with distinct substrate specificity, tissue distribution, and expression patterns (Laviad et al. 2008; Mullen et al. 2012). These isoforms catalyze the acylation of sphingoid bases with fatty acyl chains of varying lengths and degrees of saturation, generating different ceramides that are essential for many cellular processes (Gosejacob et al. 2016). CerS1 is predominantly expressed in the brain, where it is confined to neurons and skeletal muscle (Laviad et al. 2008; Mizutani et al. 2005). CerS2, which is widely distributed across various tissues, shows particularly high expression in the liver and kidneys (Laviad et al. 2008; Levy and Futerman 2010; Pewzner‐Jung et al. 2010). CerS3 is mainly involved in maintaining the skin's water permeability barrier and is highly expressed in the testis (Coderch et al. 2003; Levy and Futerman 2010; Mizutani et al. 2006). Meanwhile, it shows low expression levels in other tissues (Laviad et al. 2008). CerS4 is expressed in tissues, such as the skin, heart, liver, and leukocytes (Laviad et al. 2008). CerS5 and CerS6, which share substrate specificity generating C16‐ceramide, are expressed in various tissues, including skeletal muscle, intestine, and lymph nodes (Laviad et al. 2008; Levy and Futerman 2010).

Previous studies have demonstrated that CerS1, CerS2, and CerS4 are expressed in the mouse retina and modulate outer retinal signaling, as reflected by altered electroretinography (ERG) responses (Brüggen et al. 2016). Notably, despite these functional changes, no overt morphological abnormalities were observed in the retinas of CerS1‐, CerS2‐, and CerS4‐deficient mice, including photoreceptor (PR) number, cone outer segment length, retinal layer thickness, or synaptic organization (Brüggen et al. 2016).

Identified as the first purified mammalian CerS, CerS5 has been extensively studied for its role in the synthesis of C16‐ceramide (Levy and Futerman 2010). It reportedly exhibits low messenger RNA (mRNA) expression in most tissues (Laviad et al. 2008), with a predominant expression in pulmonary epithelial cells and detectable mRNA levels across most cells in the gray and white matter of the brain (Levy and Futerman 2010). However, despite its well‐established biochemical role, the cellular distribution and functional significance of CerS5 in the retina remain poorly understood.

Given its pivotal role in the synthesis of C16‐ceramide, a sphingolipid that promotes apoptosis, CerS5 has attracted considerable research attention (Gosejacob et al. 2016; Levy and Futerman 2010). However, its expression and role in the retina remain to be fully elucidated. Understanding the distribution, cellular localization, and functional implications of CerS5 in the retina may uncover potential mechanisms underlying retinal pathophysiology and provide new perspectives on retinal health and diseases. Therefore, the present study aimed to investigate the presence, localization, and functional significance of CerS5 in the retina using CerS5 knockout (KO) mice, with particular emphasis on its role in retinal structure and function.

## 2 Materials and Methods

### 2.1 Animals

All animal care and experimental procedures were approved by the Institutional Animal Care and Use Committee (IACUC) of Ewha Womans University (EWHA MEDIACUC 21‐043, EWHA MEDIACUC 25‐008‐t). Animals were maintained in a specific pathogen‐free barrier facility on a 12‐h light and dark cycle with free access to food and water, in accordance with institutional animal care guidelines. CerS5‐null mice (CerS5^tm2b(KOMP)Mbp^), generated by KOMP (www.KOMP.org) in a C57BL/6N background, were purchased from Jackson Laboratories (#022819; Bar Harbor, ME, USA). The knockout allele was generated by targeted insertion of a β‐galactosidase (lacZ)‐containing cassette followed by Cre‐mediated recombination, resulting in a reporter knockout allele (MGI:5490496). Genotyping was performed by PCR as previously described (Kim et al. 2022), and detailed allele information and primer sequences are provided in Table 1. A total of 56 male mice aged 2–4 months were used in this study and divided into CerS5 wild‐type (WT) and CerS5 KO groups. Some animals were used for multiple analyses; the number of animals used in each experiment is described in the respective sections. An additional six mice (three WT and three KO) aged 6 months were used for additional immunostaining and retinal thickness analysis (Figure 8). Euthanasia was performed by inhalation anesthesia using 4% isoflurane, and retinas were extracted for subsequent molecular, biochemical, and histological analyses.

**TABLE 1:** Genetic modification and genotyping information for the CerS5^tm2b(KOMP)Mbp^ allele.

Category	Description	Sequence/genomic information	Reference
Allele designation	CerS5^tm2b(KOMP)Mbp^ (C57BL/6N background)	JAX #022819	KOMP; MGI:5490496
Knockout strategy	Target insertion of an L1L2_Bact_P cassette insertion containing lacZ and neomycin resistance genes, flanked by FRT and loxP sites; critical exons deleted by Cre‐mediated recombination	Chromosome 15 insertion site: 99648826 and 99679708	MGI:5490496
Genotyping primer (WT)	Forward: 5′‐GATGGGAGTTTTTCTTTGTGC‐3′ Reverse: 5′‐TGATGGATTGGGCCTAGTGT‐3′	PCR product for wild‐type allele	Kim et al. (2022)
Genotyping primer (KO)	Forward: 5′‐CGGTCGCTACCATTACCAGT‐3′ Reverse: 5′‐TTGCCTCAAAACCCACCTAC‐3′	PCR product for null allele	Kim et al. (2022)

### 2.2 Tissue Preparation

After euthanasia by isoflurane inhalation as described in Section 2.1, tissue preparation was performed following the method of a previous study (Song et al. 2023). Briefly, the anterior segment of the eyes was removed. For immunohistochemistry, the eyecups were fixed by immersing them in 4% paraformaldehyde in 0.01 M phosphate‐buffered saline (PBS, pH 7.4) for 1.5 h. The fixed retinas were replaced with 30% sucrose overnight at 4°C. Subsequently, the samples were frozen in liquid nitrogen and stored at −80°C.

### 2.3 Real‐Time PCR

The total mRNA of the retina was extracted using the RNeasy Mini Kit (QIAGEN, Valencia, CA, USA), and complementary DNA was synthesized from the mRNA using the Verso cDNA Synthesis Kit (Thermo Fisher Scientific, Waltham, MA, USA) according to the manufacturer's instructions. Gene expression was quantified by TaqMan‐based real‐time PCR using the ABI PRISM 7500 FAST Sequence Detection System (Applied Biosystems, Foster City, CA, USA). The relative gene expression was calculated as 2*^−^* ^ΔΔCT^ in accordance with a previous study (Pfaffl 2001) and normalized with the hypoxanthine phosphoribosyltransferase (Hprt). Details of the TaqMan assay IDs and primer sequences used in this study are provided in Table 2 and were described previously (Shin et al. 2021). Retinas from four CerS5 WT and four CerS5 KO mice (*n* = 4 per group) were analyzed.

**TABLE 2:** TaqMan assay IDs and primer sequences used for real‐time PCR.

Gene	Assay/Primer	Sequence or catalog no.	Source
Hprt	TaqMan Assay	Mm00446968_m1	Applied Biosystems
CerS1	TaqMan Assay	Mm00433562_m1	Applied Biosystems
CerS2	TaqMan Assay	Mm00504086_m1	Applied Biosystems
CerS3	TaqMan Assay	Mm03990709_m1	Applied Biosystems
CerS4	TaqMan Assay	Mm01212479_m1	Applied Biosystems
CerS5	TaqMan Assay	Mm00510996_g1	Applied Biosystems
CerS6	TaqMan Assay	Mm00556165_m1	Applied Biosystems
Gata3	Forward: 5′‐CAG CTG CCA GAT AGC ATG AA‐3′ Reverse: 5′‐CAT AGG GCG GAT AGG TGG TA‐3′	Custom primer	Shin et al. (2021)
RORγt	Forward: 5′‐GGA GCT CTG CCA GAA TGA GC‐3′ Reverse: 5′‐CAA GGC TCG AAA CAG CTC CAC‐3′	Custom primer	Shin et al. (2021)

### 2.4 Ceramide Quantification by Liquid Chromatography–Electrospray Ionization–Tandem Mass Spectrometry (LC–ESI–MS/MS)

Ceramide levels were analyzed using LC–ESI–MS/MS. Total lipids were extracted from retinal tissues collected from three CerS5 WT and three CerS5 KO mice (*n* = 3 per group) and injected into an ExionLC Series UHPLC (AB SCIEX, Toronto, Canada). Lipid separation was performed using a Kinetex HILIC column (2.1 × 100 mm^2^, 2.6 µm ID; Phenomenex, St. Louis, MO, USA). The eluted lipids were subsequently analyzed using an API 5500 QTRAP mass spectrometer (AB SCIEX) operated in positive ESI mode. Data acquisition and processing were conducted using Analyst 1.6.3 software (Applied Biosystems).

### 2.5 Semithin Sections

The superior temporal region of the retinas was processed as described by Song et al. (2023). Briefly, dehydrated retinas were embedded in epoxy resin, sectioned at 400‐nm‐thick, stained with toluidine blue, and mounted. The slides were imaged using the BX‐50 light microscope (Olympus, Tokyo, Japan). Retina from two CerS5 WT and two CerS5 KO mice (2–4 months of age, *n* = 2 per group) were used for semithin sections analysis.

### 2.6 Frozen Section and Retinal Thickness Measurement

After fixation and cryoprotection, the eyecups were retrieved, thawed, embedded in optimal cutting temperature (OCT) compound (Sakura Finetek USA, Torrance, CA, USA), and frozen. Frozen sections (20 µm thick) were obtained using a cryostat microtome (CM 1850, Leica Biosystems, Nussloch, Germany) and mounted on glass slides using mounting medium containing DAPI (Vector Laboratories, Newark, CA, USA). Frozen sections passing through or near the optic nerve head were selected for analysis. Retinal thickness was measured in these sections under a light microscope by averaging measurements from at least five different points per section. For this analysis, retinal tissues from three CerS5 WT and three CerS5 KO mice (2–4 months of age, *n* = 3 per group) were used. Retinal thickness was additionally measured in three 6‐month‐old WT and three 6‐month‐old KO mice (*n* = 3 per group) to assess potential age‐related structural changes (Figure 8A).

### 2.7 Terminal Deoxynucleotidyl Transferase dUTP Nick End Labeling (TUNEL) Assay

To determine the number of dead cells, TUNEL fluorescence assay was performed by partially modifying the protocol enclosed in the FragEL DNA Fragmentation Detection Kit (QIA39, MilliporeSigma, Burlington, MA, USA). The vibratome‐sectioned retinas stained with X‐gal were incubated in TdT equilibration buffer for 20 min at room temperature. Thereafter, the TdT‐labeled reaction mixture reacted at room temperature for 1 h, and then the vibratome‐sectioned retinas were mounted on slides using VECTASHIELD with DAPI H‐1200 (Vector Laboratories). The retinas from one postnatal Day 0 (P0) mouse were obtained and used as a positive control in the TUNEL assay. All TUNEL‐stained sections were imaged using confocal microscopy (LSM800, Carl Zeiss, Oberkochen, Germany) to visualize apoptotic cells and assess colocalization with X‐gal signals.

### 2.8 X‐Gal Staining

X‐gal staining was performed in accordance with the method of Brüggen et al. (2016). Retinal tissues were sectioned at 50 µm thickness using a vibratome and were incubated in LacZ washing solution (2 mM magnesium chloride (MgCl_2_), 5 mM ethylene glycol‐bis (β‐aminoethyl ether)‐*N,N,N′,N′*‐tetraacetic acid (EGTA, pH 8.0), 0.01% sodium deoxycholate, 2% Nonidet P‐40, and 0.01 M PBS) for 15 min at room temperature. Then, the sections were incubated overnight at 37°C in LacZ substrate solution (2 mM MgCl_2_, 5 mM EGTA (pH 8.0), 5 mM potassium ferricyanide (K_3_[Fe(CN)_6_]), 5 mM potassium ferrocyanide (K_4_[Fe(CN)_6_]), 0.01 M PBS, and 1 mg/mL X‐gal stock solution). Afterward, the sections were washed and mounted using VECTASHIELD with H‐1000 solution (Vector Laboratories). Some of the stained sections were imaged using light microscopy (BX‐50, Olympus, Tokyo, Japan) to visualize X‐gal staining, whereas others were processed for a subsequent immunohistochemical step. Quantification of X‐gal staining intensity was performed using ImageJ software (National Institutes of Health [NIH], Bethesda, MD, USA) by measuring the stained areas.

### 2.9 Immunohistochemistry

For the immunohistochemical staining, X‐gal‐stained retinal sections were blocked with 10% normal serum and incubated overnight at 4°C with primary antibodies (Table 3) diluted in PBS. After washing, sections were incubated for 2 h at room temperature with secondary antibodies; Alexa Fluor 488–conjugated goat anti‐mouse IgG (1:200, cat no. A11017; Invitrogen, Waltham, MA, USA), Cy3‐conjugated donkey anti‐mouse IgG (1:500, cat no. 715‐166‐151; Jackson ImmunoResearch, West Grove, PA, USA), or Cy3‐conjugated goat anti‐rabbit IgG (1:500, cat no. 111‐166‐003; Jackson ImmunoResearch). Slides were mounted with VECTASHIELD with DAPI (Vector Laboratories) and imaged using an LSM800 confocal microscope (Carl Zeiss). Images were uniformly processed with ZEN software (Carl Zeiss). For immunohistochemistry, WT (*n* = 10) and KO (*n* = 11) retinas were analyzed. Additional immunostaining was performed in retinas from three WT and three KO mice aged 6 months (*n* = 3 per group) to evaluate the consistency of CerS5 expression patterns at a later age (Figure 8B,C).

**TABLE 3:** List of primary antibodies used for immunohistochemistry.

Antibody	Target cell	Dilution	Manufacturer	Cat no.
Mouse anti‐calbindin	Horizontal cells	1:2000	Sigma‐Aldrich	C9848
Goat anti‐ChAT	Cholinergic amacrine cells	1:300	Merck Millipore	AB144P
Rabbit anti‐GABA	GABAergic amacrine cells	1:3000	Sigma‐Aldrich	A2052
Mouse anti‐GS	Müller cells	1:4000	Merck Millipore	MAB302
Rabbit anti‐PKCα	Rod bipolar cells	1:1000	Santa Cruz	SC‐208
Rabbit anti‐RBPMS	Ganglion cells	1:1000	Merck Millipore	ABN1362
Mouse anti‐SMI‐32	Alpha ganglion cells	1:500	Merck Millipore	NE1023
Rabbit anti‐TH	Dopaminergic amacrine cells	1:1000	Merck Millipore	AB152

### 2.10 Soma Density Estimation

Soma density was estimated from vibratome‐sectioned retinas processed for immunohistochemistry as described in Section 2.9. DAPI‐positive nuclei were counted within a fixed region of interest (ROI) in each retinal layer: approximately 1178 µm^2^ (220 × 220 pixels) for the outer nuclear layer (ONL), 789 µm^2^ (180 × 180 pixels) for the inner nuclear layer (INL), and 1093 µm^2^ (500 × 90 pixels) for the ganglion cell layer (GCL). The elongated ROI for the GCL reflects the sparse cell distribution in this layer. The same ROI dimensions were applied to both WT and KO sections, and results were expressed as estimated cells/mm^2^. It should be noted that the ROI height for the GCL was determined on the basis of CerS5 KO retinal thickness; as WT GCL thickness was greater, soma density in WT retinas may be slightly underestimated. Retinal tissues from eight CerS5 WT and eight CerS5 KO mice (2–4 months of age, *n* = 8 per group) were analyzed.

### 2.11 ERG Analysis

ERG was performed in accordance with the method of Kim et al. (2019). Mice were dark‐adapted overnight and then anesthetized with a mixture of zolazepam (30 mg/kg) and xylazine (10 mg/kg). Body temperature was maintained at 35–37°C using a heating pad. The ERG recordings were performed under dim red light (*λ* > 600 nm). After positioning the mouse, hydroxypropyl methylcellulose was applied to the cornea, and the active electrodes of the gold ring were covered. The ground and reference electrodes were placed subcutaneously in the neck and tail, respectively. Scotopic ERG was recorded using a Ganzfeld stimulator (UTAS BigShot, LKC Technologies, Gaithersburg, MD, USA) with white‐light stimuli intensities ranging from −2 to 1.3 log cd s/m^2^. Each stimulus repeated three times at 15‐s intervals to calculate the average response, with the band‐pass filter range between 0.3 and 300 Hz for all scotopic ERG recordings. The ERG data were analyzed using the UTAS software provided by the manufacturer. ERG was performed on WT (*n* = 7) and KO (*n* = 8) mice at 2–4 months of age. a‐wave amplitudes are presented as absolute values of the negative deflection. Photopic ERG was not performed in this study.

### 2.12 Statistical Analysis

All statistical analyses were performed using GraphPad Prism (GraphPad Software, La Jolla, CA, USA). For comparison between two groups, statistical significance was analyzed using unpaired Student's *t*‐tests. For lipidomic data, multiple comparisons were controlled using the false discovery rate according to the original method of Benjamini and Hochberg. For multiple comparisons in ERG data, the Bonferroni–Dunn correction was applied to control for Type I error. Statistical significance was considered at *p* < 0.05. The analytical data are presented as the mean ± standard error of the mean.

## 3 Results

### 3.1 *CerS5* Gene Deletion Validation by Real‐Time PCR and Ceramide Profiling by Lipidomic

To determine whether the *CerS5* gene was properly deleted in CerS5 KO mouse retinas, we quantified the relative mRNA expression levels of six CerS isoforms (CerS1–CerS6) in CerS5 WT and KO mice using real‐time PCR. The expression levels were normalized to CerS1 expression in CerS5 WT retinas.

**FIGURE 1:**
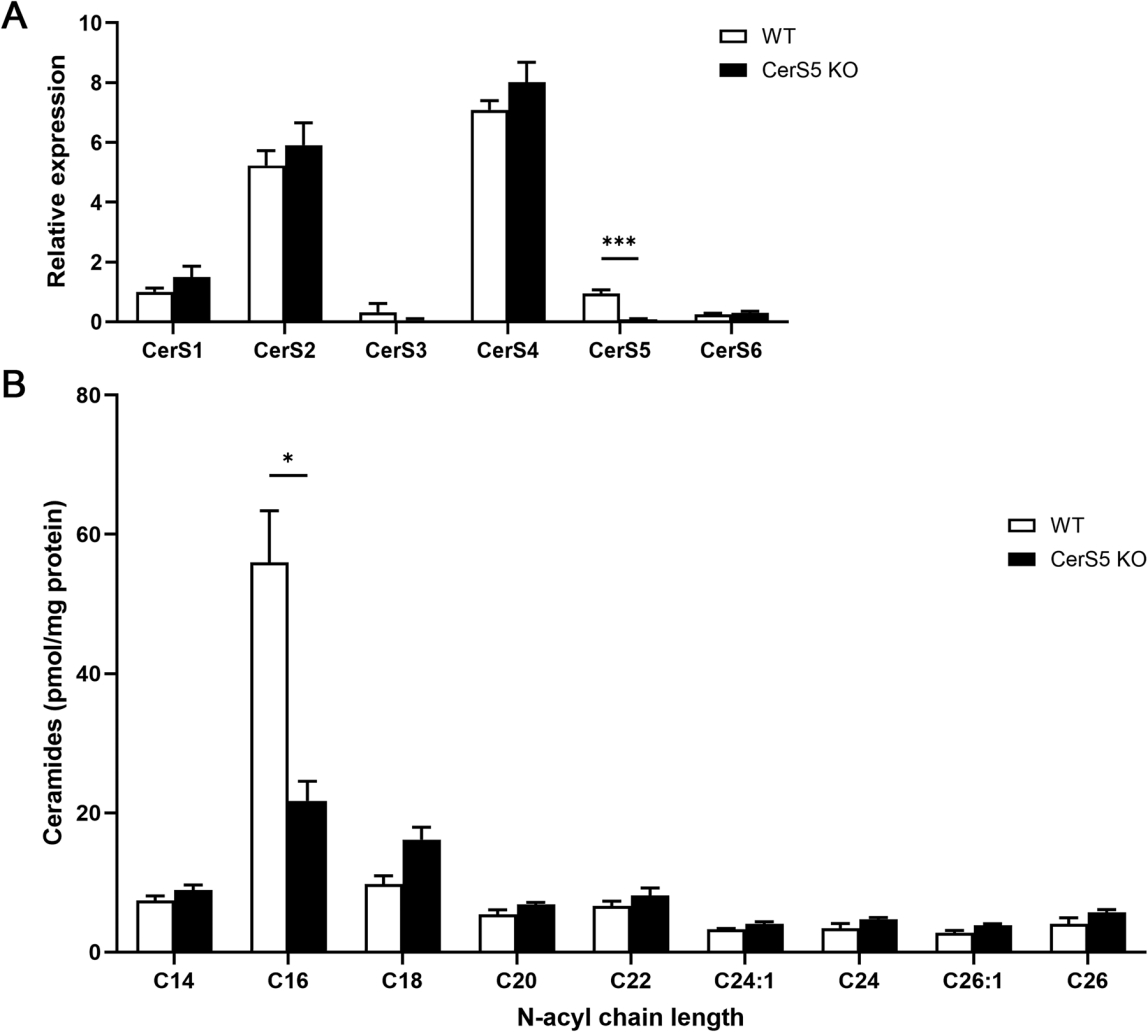
Relative mRNA expression of ceramide synthase (CerS) isoforms (A) and lipidomic analysis (B) in CerS5 wild‐type (WT) and knockout (KO) mouse retinas. All CerS isoforms (CerS1 to CerS6) were expressed in both CerS5 WT and KO retinas. The expression levels of CerS1, CerS2, CerS4, and CerS6 were slightly higher in CerS5 KO retinas, whereas CerS3 was slightly lower; however, none of these changes were statistically significant. Only CerS5 expression was significantly decreased in KO retinas (A). Lipidomic analysis showed that C16‐ceramide levels were significantly decreased in CerS5 KO retinas compared with CerS5 WT retinas. Although C18‐ceramide levels appeared increased, this change did not reach statistical significance after correction for multiple comparisons. No significant differences were observed in other ceramide *N*‐acyl chain length (B). Differences were significant at *p < 0.05 and ***p < 0.001.

Among these isoforms, only CerS5 mRNA was significantly reduced in CerS5 KO retinas compared to CerS5 WT (0.94 ± 0.13 in CerS5 WT vs. 0.09 ± 0.02 in CerS5 KO, *p* < 0.001), confirming the selective deletion of CerS5. The mRNA expression levels of CerS1 (1.00 ± 0.13 in CerS5 WT vs. 1.49 ± 0.39 in CerS5 KO, *p* = 0.26), CerS2 (5.23 ± 0.50 vs. 5.91 ± 0.74, *p* = 0.47), CerS4 (7.09 ± 0.31 vs. 8.01 ± 0.67, *p* = 0.26), and CerS6 (0.25 ± 0.04 vs. 0.29 ± 0.06, *p *= 0.57) were slightly higher in CerS5 KO retinas, whereas CerS3 expression (0.32 ± 0.29 vs. 0.05 ± 0.05, *p* = 0.35) was slightly lower. However, none of these differences were statistically significant (*p* > 0.05 for all) (Figure 1A).

In addition to gene expression, we performed lipidomic analysis to examine whether CerS5 deficiency affected retinal ceramide composition. Consistent with the known role of CerS5 in C16‐ceramide biosynthesis, C16‐ceramide levels were significantly reduced in CerS5 KO retinas compared to CerS5 WT retinas (55.95 ± 7.45 vs. 21.74 ± 2.81, *p* = 0.01). In contrast, C18‐ceramide levels showed an apparent increase (9.75 ± 1.22 vs. 16.18 ± 1.79, *p* = 0.04); however, this difference did not remain statistically significant after correction for multiple comparisons. Ceramides with other *N*‐acyl chain lengths showed no significant changes (*p* > 0.05), although most observed a trend toward mild elevation in CerS5 KO retinas that did not reach statistical significance (Figure 1B).

### 3.2 Morphology of CerS5 KO Retinas

**FIGURE 2:**
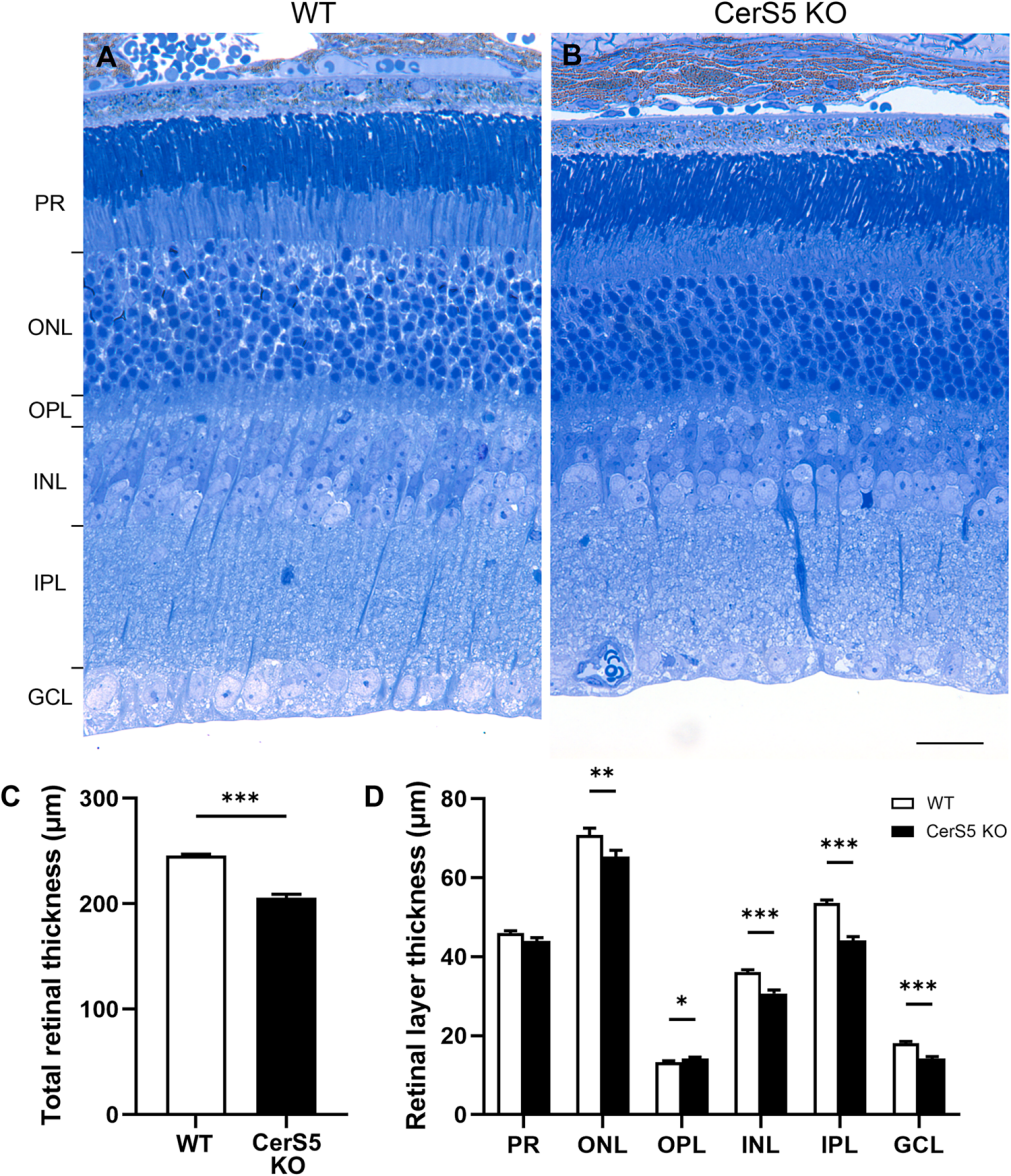
Images of 400‐nm semithin sections stained with toluidine blue from CerS5 wild‐type (WT) (A) and knockout (KO) retinas (B). No obvious morphological differences were observed between CerS5 WT and KO retinas, except for a reduction in overall retinal thickness. The total retinal thickness was significantly decreased in CerS5 KO retinas than CerS5 WT retinas (C). Layer‐specific analysis revealed that the ONL, INL, IPL, and GCL were significantly thinner in CerS5 KO retinas than in CerS5 WT retinas. The PR thickness did not differ significantly between CerS5 WT and KO retinas, whereas the OPL was significantly thicker in CerS5 KO retinas (D). Differences were significant at *p < 0.05, **p < 0.01, and ***p < 0.001. Scale bar = 50 µm in (A) and (B). GCL, ganglion cell layer; INL, inner nuclear layer; IPL, inner plexiform layer; ONL, outer nuclear layer; OPL, outer plexiform layer; PR, photoreceptor.

To determine whether CerS5 deficiency affects retinal morphology, semithin retinal sections were stained with toluidine blue. No apparent morphological abnormalities were observed in CerS5 KO retinas compared to CerS5 WT retinas, and the overall laminar structure appeared to be preserved (Figure 2A,B).

To quantitatively assess retinal thickness, measurements were performed using DAPI‐stained frozen sections. Total retinal thickness was significantly reduced in CerS5 KO retinas (245.50 ± 1.53 µm in CerS5 WT vs. 205.74 ± 3.42 µm in CerS5 KO, *p* < 0.001), representing a reduction of approximately 16.20% (Figure 2C). Layer‐specific analysis revealed significant thinning in most neural retinal layers of CerS5 KO mice, including the ONL (70.85 ± 1.73 vs. 65.35 ± 1.62 µm, *p* = 0.01), INL (36.08 ± 0.65 vs. 30.71 ± 0.84 µm, *p* < 0.001), inner plexiform layer (IPL: 53.62 ± 0.73 vs. 44.13 ± 0.95 µm, *p* < 0.001), and GCL (18.13 ± 0.45 vs. 14.26 ± 0.41 µm, *p* < 0.001). The PR thickness showed a mild reduction (45.99 ± 0.55 vs. 43.95 ± 0.88 µm), but the difference was not statistically significant (*p* = 0.06). In contrast, the outer plexiform layer (OPL) was the only layer that exhibited a significant increase in thickness, approximately 7.23% thicker in CerS5 KO retinas than in WT retinas (13.30 ± 0.34 vs. 14.26 ± 0.31 µm, *p* = 0.04) (Figure 2D).

### 3.3 CerS5 Expression in Specific Locations in Mouse Retina

**FIGURE 3:**
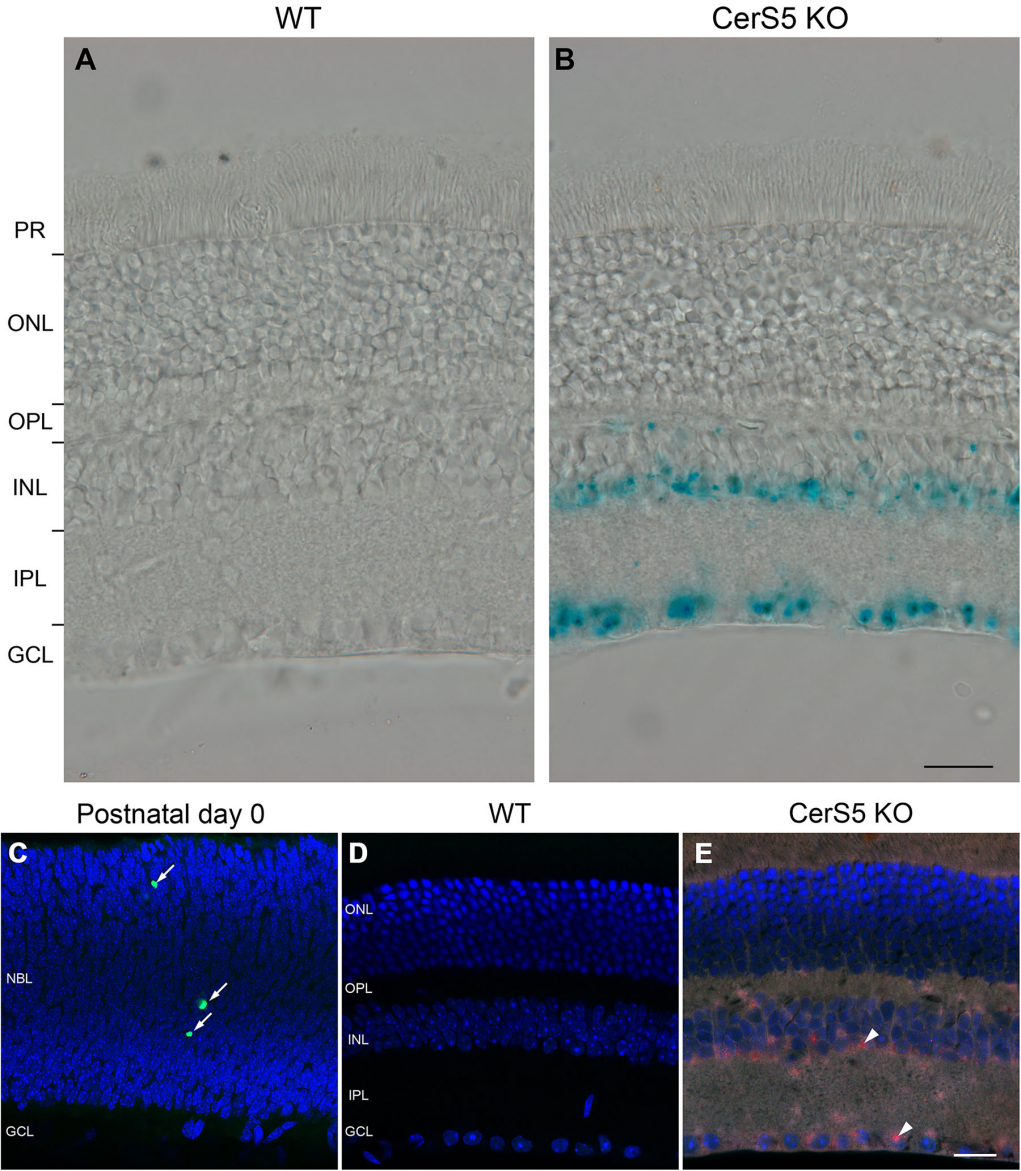
Images of vertical vibratome retinal sections stained with X‐gal (A and B) and terminal deoxynucleotidyl transferase dUTP nick end labeling (TUNEL) assay combined with X‐gal staining and DAPI counterstaining (C–E) from CerS5 wild‐type (WT) and knockout (KO) mice. Vertical vibratome retinal sections stained with X‐gal from CerS5 WT and KO mice. *lacZ* was inserted into the *CerS5* gene locus, allowing β‐galactosidase expression, which was confirmed by X‐gal staining. No blue staining was detected in CerS5 WT retinas (A), whereas partial blue staining was observed in CerS5 KO retinas (B). In CerS5 KO retinas, X‐gal positive signals were localized specifically to the INL and GCL (B). TUNEL staining (green, arrows) was performed to detect apoptotic cells, combined with X‐gal staining (red, arrowheads) indicating LacZ expression, and DAPI counterstaining (blue) to visualize cell nuclei. TUNEL‐positive cells (green, arrows) were detected in the postnatal Day 0 retina, which served as a positive control (C). No TUNEL‐positive cells were detected in WT (D) or KO (E) retinas. In CerS5 KO retinas, only X‐gal staining was observed without evidence of apoptosis. Scale bar = 50 µm in (A) and (B); 20 µm in (C–E). GCL, ganglion cell layer; INL, inner nuclear layer; IPL, inner plexiform layer; NBL, neuroblast cell layer; ONL, outer nuclear layer; OPL, outer plexiform layer; PR, photoreceptor.

To determine the distribution of the *CerS5* gene loci in retinal tissue, we utilized the LacZ reporter from the L1L2_Bact_P cassette. CerS5 localization in CerS5 KO retinas was visualized by using X‐gal staining, which reflects β‐galactosidase activity driven by the lacZ reporter. No detectable X‐gal signal was observed in the CerS5 WT retina (Figure 3A). By contrast, strong blue staining was observed in CerS5 KO retinas using light microscopy (Figure 3B). This staining pattern indicated that *CerS5* is specifically localized to the INL and GCL in CerS5 KO retinas.

**FIGURE 4:**
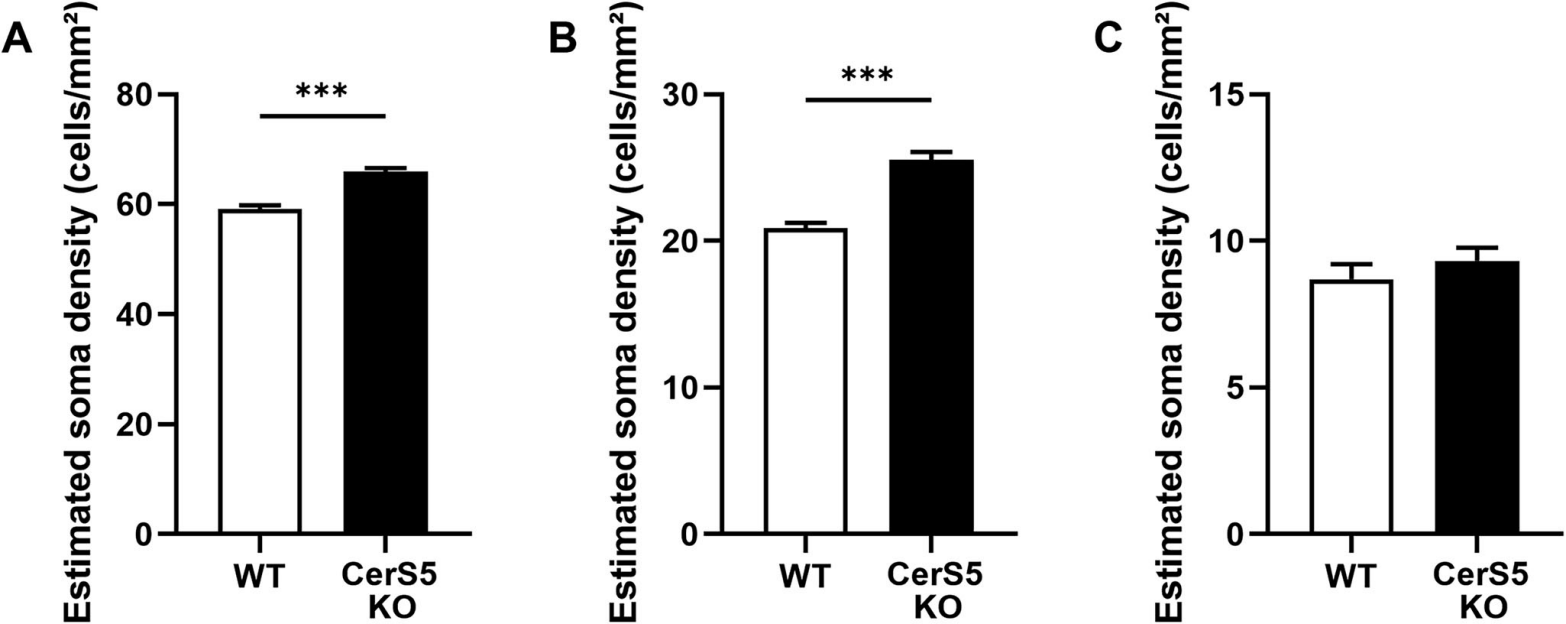
Estimated soma density in the ONL, INL, and GCL of 2–4‐month‐old CerS5 wild‐type (WT) and knockout (KO) mouse retinas. DAPI‐positive nuclei were counted within a fixed ROI from vibratome sections used for immunohistochemistry and expressed as estimated cells/mm^2^. Soma density was significantly increased in the ONL (A) and INL (B) of CerS5 KO retinas, whereas no significant difference was detected in the GCL (C). Differences were significant at ***p < 0.001. GCL, ganglion cell layer; INL, inner nuclear layer; ONL, outer nuclear layer.

**FIGURE 5:**
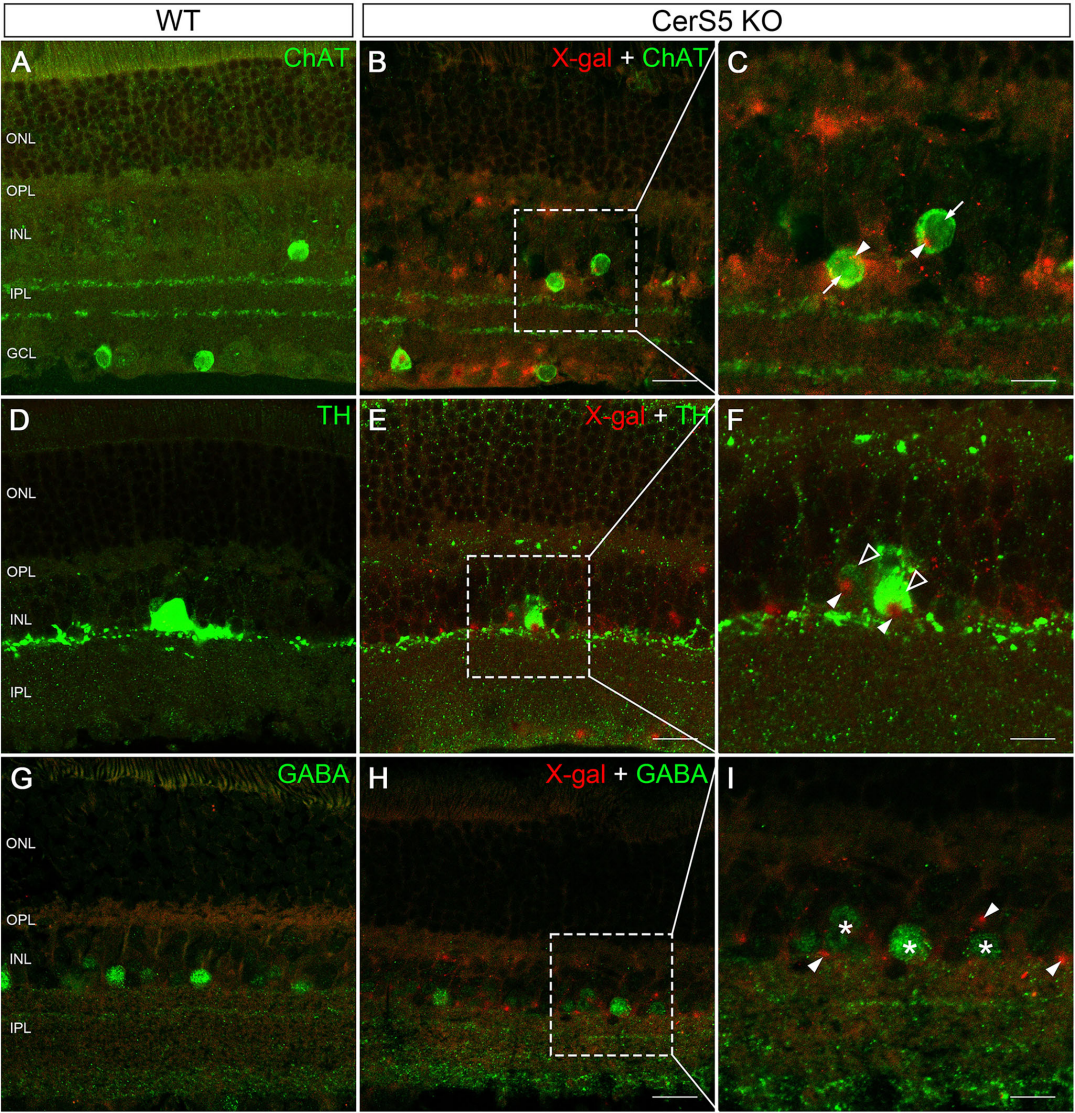
Vertical vibratome‐sectioned retinas from CerS5 wild‐type (WT) and knockout (KO) mice immunolabeled with amacrine cell markers and stained with X‐gal. The nuclei of cholinergic (arrows, A–C), dopaminergic (open arrowheads, D–F), and GABAergic (asterisks, G–I) amacrine cells (green) were mainly localized to the INL, with some cells also present in the GCL. ChAT‐ and TH‐positive cells colocalized with X‐gal signals (arrowheads, red), whereas GABA‐positive cells did not colocalize with X‐gal. Scale bars = 20 µm in (A), (B), (D), (E), (G), and (H); 10 µm in (C), (F), and (I). ChAT, choline acetyltransferase; GABA, γ‐aminobutyric acid; GCL, ganglion cell layer; INL, inner nuclear layer; IPL, inner plexiform layer; ONL, outer nuclear layer; OPL, outer plexiform layer; TH, tyrosine hydroxylase.

**FIGURE 6:**
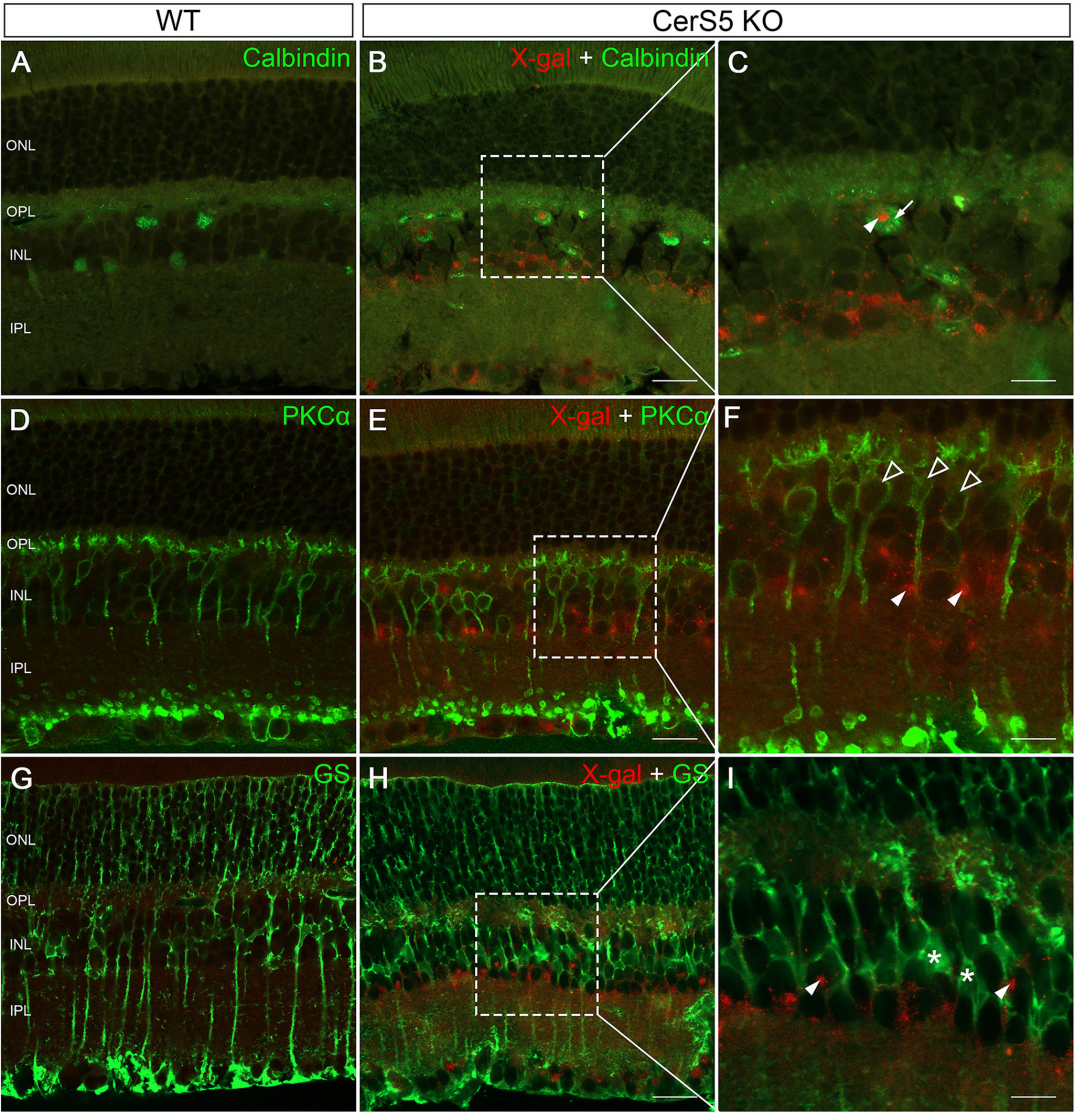
Immunostaining of vertical retinal sections from CerS5 wild‐type (WT) and knockout (KO) mice using cell‐specific markers expressed in the INL and double‐stained with X‐gal. The cellular markers labeled in green are calbindin (arrows, A–C), PKCα (open arrowheads, D–F), and GS (asterisks, G–I), all of which are expressed in the INL. Colocalization of X‐gal staining (arrowheads, red) was observed only in calbindin‐positive cells. Cells immunostained for PKCα and GS showed no colocalization with the X‐gal signals. Scale bars = 20 µm in (A), (B), (D), (E), (G), and (H); 10 µm in (C), (F), and (I). GS, glutamine synthetase; INL, inner nuclear layer; IPL, inner plexiform layer; ONL, outer nuclear layer; OPL, outer plexiform layer; PKCα, protein kinase C alpha.

To further verify this localization, confocal microscopy was used to detect X‐gal–associated signals, as X‐gal precipitates can be visualized under 633 nm laser excitation (Levitsky et al. 2013). Confocal images acquired in the Cy5 channel revealed X‐gal signals exclusively in CerS5 KO retinas, with no specific signal detected in CerS5 WT retinas (Figures 3, 4, 5, 6). Because CerS5 is an essential membrane‐associated protein involved in ceramide synthesis in the endoplasmic reticulum (Levy and Futerman 2010; Stiban et al. 2010), its expression in the retina was, as expected, restricted to the cytoplasm and did not localize to the nucleus (Figures 3, 4, 5, 6).

### 3.4 Absence of Cell Loss Despite Retinal Thinning in CerS5 KO Mice

To determine whether CerS5 deficiency influences retinal cell death, we performed TUNEL assays on the retinas from CerS5 WT and CerS5 KO mice. Retinal tissue from P0 mice, where cell death predominantly occurs in the inner retinal layers (Beckmann et al. 2021), was used as a positive control to validate the assay. As expected, TUNEL‐positive cells were detected in the P0 retina (Figure 3C). However, no apoptotic cells were observed in the retinas of either CerS5 WT or CerS5 KO mice (Figure 3D,E).

To further assess whether cell number was preserved, soma density was estimated from DAPI‐stained vibratome sections in the ONL, INL, and GCL of 2–4‐month‐old mice. Soma density was significantly higher in the ONL (59.15 ± 0.72 cells/mm^2^ in WT vs. 65.97 ± 0.62 cells/mm^2^ in KO, *p* < 0.001) and INL (20.85 ± 0.37 vs. 25.55 ± 0.52 cells/mm^2^, *p* < 0.001) of CerS5 KO retinas compared to WT retinas. In the GCL, soma density showed a trend toward increase in CerS5 KO retinas but did not reach statistical significance (8.69 ± 0.52 vs. 9.31 ± 0.45 cells/mm^2^, *p* = 0.37) (Figure 4).

### 3.5 Colocalization of CerS5 With Amacrine Cells

To determine which retinal cell types exhibited X‐gal signals, we performed immunolabeling using various amacrine cell markers localized to the INL and GCL (Figure 5). The nuclei of most amacrine cells are primarily located in the INL, with neuronal dendrite processes extending toward the IPL (Balasubramanian and Gan 2014; Kolb 1997). Some amacrine cells, known as displaced amacrine cells, have their nuclei located in the GCL (Balasubramanian and Gan 2014). Amacrine cells can be classified into cholinergic, dopaminergic, and GABAergic subtypes on the basis of their neurotransmitters (Pourcho 1996).

In cholinergic amacrine cells immunolabeled with choline acetyltransferase (ChAT) (Haverkamp and Wassle 2000; Jeon et al. 1998; Rodieck and Marshak 1992), nuclei were observed in both the INL and GCL, and their dendrites formed two distinct bands within the IPL (Millar et al. 1987; Xu et al. 2003). In ChAT‐labeled Cers5 WT and KO retinas, the nuclei of cholinergic amacrine cells were located in the INL and GCL (Figure 5A–C). In CerS5 KO retinas, X‐gal signals (visualized as Cy5 fluorescence) colocalized with the cytoplasm of cholinergic amacrine cells (Figure 5B,C), whereas only ChAT‐positive cells were detected in CerS5 WT retinas (Figure 5A).

Dopaminergic amacrine cells, identified by tyrosine hydroxylase‐immunoreactivity (TH‐IR), showed nuclei were primarily located in the INL, with occasional cells observed in the GCL (Ballesta et al. 1984; Dacey 1990). Their dendrites ran parallel to the INL (Haverkamp and Wassle 2000). Both CerS5 WT and KO retinas showed TH‐IR‐positive cells in the INL (Figure 5D–F). In CerS5 KO retinas, partial colocalization of X‐gal staining with TH‐positive cells was observed (Figure 5E,F), whereas no X‐gal staining was detected in CerS5 WT retinas (Figure 5D).

GABAergic amacrine cells, immunolabeled for γ‐aminobutyric acid (GABA), were primarily localized to the INL (Pow et al. 1995), and their dendrites formed two distinct bands in the IPL, similar to cholinergic amacrine cells (Haverkamp and Wassle 2000; Vaney and Young 1988). In both CerS5 WT and KO retinas, GABAergic amacrine cell nuclei were detected in the INL. Notably, no colocalization between X‐gal signals and GABA‐positive cells was observed in either CerS5 WT or KO retinas (Figure 5G–I).

### 3.6 Colocalization of CerS5 With Other Retinal Cells in the INL

To confirm whether X‐gal signals colocalize with other retinal cell types located in the INL, we performed immunostaining with specific cell markers. In addition to amacrine cells, the INL contained the nuclei of horizontal cells, rod bipolar cells, and Müller glial cells (Masri et al. 2021).

Horizontal cells are responsible for transmitting visual information from PRs to bipolar cells (Chapot et al. 2017; Chaya et al. 2017). Calbindin, a marker for horizontal cells, was detected in the somata, dendrites, and axons of horizontal cells within the INL (Haverkamp and Wassle 2000). In both CerS5 WT and KO retinas, horizontal cells were immunolabeled with calbindin. In CerS5 KO retinas, colocalization of X‐gal signals with calbindin‐positive cells was observed (Figure 6A–C).

Rod bipolar cells relay signals from PRs to retinal ganglion cells (RGCs) (Kaneko 1983). Protein kinase C alpha (PKCα), a marker for rod bipolar cells, was expressed along the nuclear membrane of numerous bipolar cells in the INL, with processes extending into the IPL (Haverkamp et al. 2003). PCKα immunoreactivity was observed in both CerS5 WT and KO retinas. However, X‐gal signals in CerS5 KO retinas did not colocalize with PKCα‐positive rod bipolar cells (Figure 6D–F).

The nuclei of Müller glia cells, which provide structural support to retinal neurons, are located in the INL, with processes spanning from the ONL to the GCL (Bringmann et al. 2006). These processes branch into fine extensions within the OPL and IPL (Chao et al. 1997; Vecino et al. 2016; Wang et al. 2017). In mouse retina, Müller cells are immunoreactive for glutamine synthetase (GS) (Germer et al. 1997; Riepe and Norenburg 1977). Müller cells in both CerS5 WT and KO retinas were labeled with GS; however, X‐gal staining in CerS5 KO retinas did not colocalize with GS‐positive Müller cells (Figure 6G–I).

### 3.7 Colocalization of CerS5 With RGCs

To examine whether RGCs in the GCL colocalize with X‐gal signals, we performed immunostaining using established RGC markers. RGCs process visual information received from bipolar and amacrine cells and transmit it to the brain (Kim et al. 2021; Zhang et al. 2012). RNA‐binding protein with multiple splicing (RBPMS) and non‐phosphorylated neurofilament H (SMI‐32) antibodies were used as markers for general RGCs and alpha RGCs, respectively, to further characterize RGC populations and their specific subtypes (Kwong et al. 2010; Rodriguez et al. 2014). Although the majority of RGC nuclei are confined to the GCL, a small subset of displaced RGCs can be found in the INL (Kim et al. 2021; Kwong et al. 2010).

**FIGURE 7:**
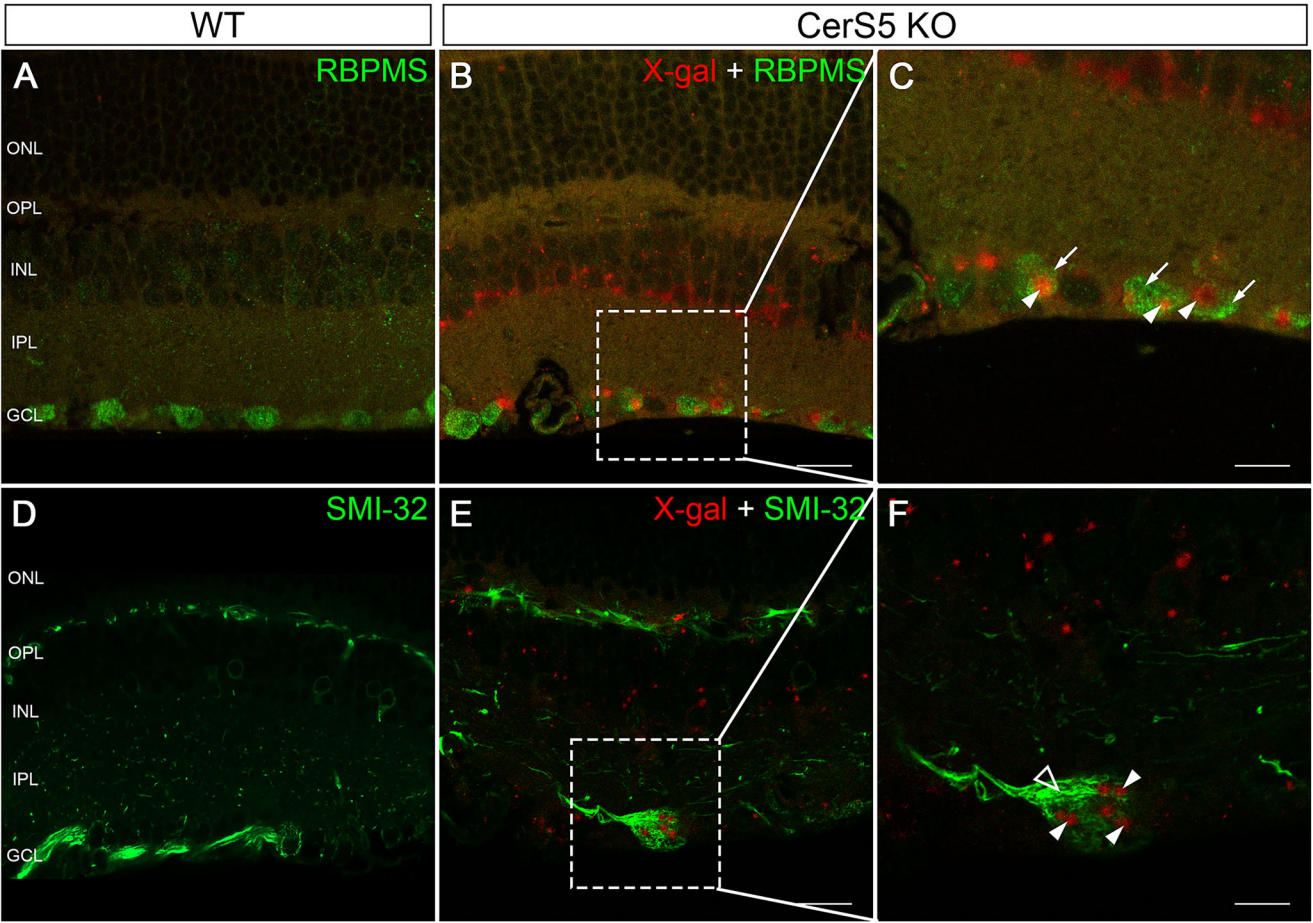
Confocal images of vertical retinal sections from CerS5 wild‐type (WT) and knockout (KO) mice immunolabeled with ganglion cell markers and co‐stained with X‐gal. Retinal ganglion cells were labeled in green with RBPMS (arrows, A–C) or SMI‐32 (open arrowheads, D–F), with most nuclei located in the GCL. Colocalization of X‐gal signals (arrowheads, red) with both RBPMS‐positive and SMI‐32‐positive ganglion cells was observed in CerS5 KO retinas. However, colocalization was limited to a subset of immunolabeled ganglion cells was observed in CerS5 KO retinas. Scale bars = 20 µm in (A), (B), (D), and (E); 10 µm in (C) and (F). GCL, ganglion cell layer; INL, inner nuclear layer; IPL, inner plexiform layer; ONL, outer nuclear layer; OPL, outer plexiform layer; RBPMS, RNA‐binding protein with multiple splicing; SMI‐32, non‐phosphorylated neurofilament H.

RBPMS‐positive RGCs were detected within the GCL in both CerS5 WT and KO retinas. In CerS5 KO retinas, RBPMS‐positive cells colocalized with X‐gal signals, suggesting that CerS5 is expressed in a subset of RGCs (Figure 7A–C).

SMI‐32 immunoreactivity, marking alpha RGCs, was observed within the GCL of both CerS5 WT and KO retinas. SMI‐32 specifically labels the non‐phosphorylated neurofilament heavy chain, which is enriched in alpha RGCs and reflects their characteristic neurofilament distribution (Straznicky et al. 1992). In CerS5 KO retinas, partial colocalization of X‐gal signals with SMI‐32‐positive alpha RGCs was observed in the GCL (Figure 7D–F).

### 3.8 CerS5 Expression and Retinal Structure in 6‐Month‐Old Mice

**FIGURE 8:**
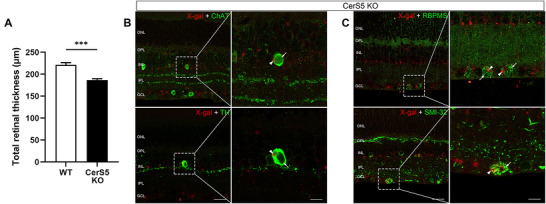
Total retinal thickness and immunostaining of vertical sections from 6‐month‐old CerS5 wild‐type (WT) and knockout (KO) mice. Total retinal thickness was significantly decreased in CerS5 KO retinas compared to age‐matched WT retinas (A). Colocalization of X‐gal signals (red, arrowheads) with ChAT‐positive cholinergic amacrine cells and TH‐positive dopaminergic amacrine cells (green, arrows) was observed in CerS5 KO retinas (B). Colocalization of X‐gal signals (red, arrowheads) with RBPMS‐positive and SMI‐32‐positive retinal ganglion cells (green, arrows) was also observed in CerS5 KO retinas (C). These patterns were consistent with those observed in 2–4‐month‐old mice. Differences were significant at ***p < 0.001. Scale bars = 20 µm in the left panels of (B) and (C), 10 µm in the right panels of (B) and (C). ChAT, choline acetyltransferase; GCL, ganglion cell layer; INL, inner nuclear layer; IPL, inner plexiform layer; ONL, outer nuclear layer; OPL, outer plexiform layer; RBPMS, RNA‐binding protein with multiple splicing; SMI‐32, non‐phosphorylated neurofilament H; TH, tyrosine hydroxylase.

To determine whether the structural changes and cellular localization of CerS5 observed in young adult mice were maintained with age, retinal thickness measurements and immunostaining were additionally performed in 6‐month‐old CerS5 WT and KO mice. Total retinal thickness remained significantly reduced in CerS5 KO retinas compared to age‐matched WT retinas (220.98 ± 5.44 µm in CerS5 WT vs. 186.32 ± 3.37 µm in CerS5 KO, *p* < 0.001), corresponding to a 15.68% decrease, consistent with the magnitude of thinning observed in younger animals (Figure 8A).

In 6‐month‐old CerS5 retinas, colocalization between X‐gal signals and retinal cell markers, including ChAT‐ and TH‐positive amacrine cells and RBPMS‐ and SMI‐32‐positive RGCs, was consistently observed, similar to the patterns found in 2–4‐month‐old mice (Figure 8B,C).

### 3.9 Retinal Function Is Unaltered in CerS5 KO Mice

ERGs recordings were performed in CerS5 WT and KO mice to assess the effects of CerS5 deficiency on retinal function. ERG measures the electrical response of the retina to light stimulation (McCulloch et al. 2015), with the a‐wave reflecting PR activity and the b‐wave representing the function of bipolar and Müller cells (Bush and Sieving 1994; Newman and Odette 1984; Stockton and Slaughter 1989).

**FIGURE 9:**
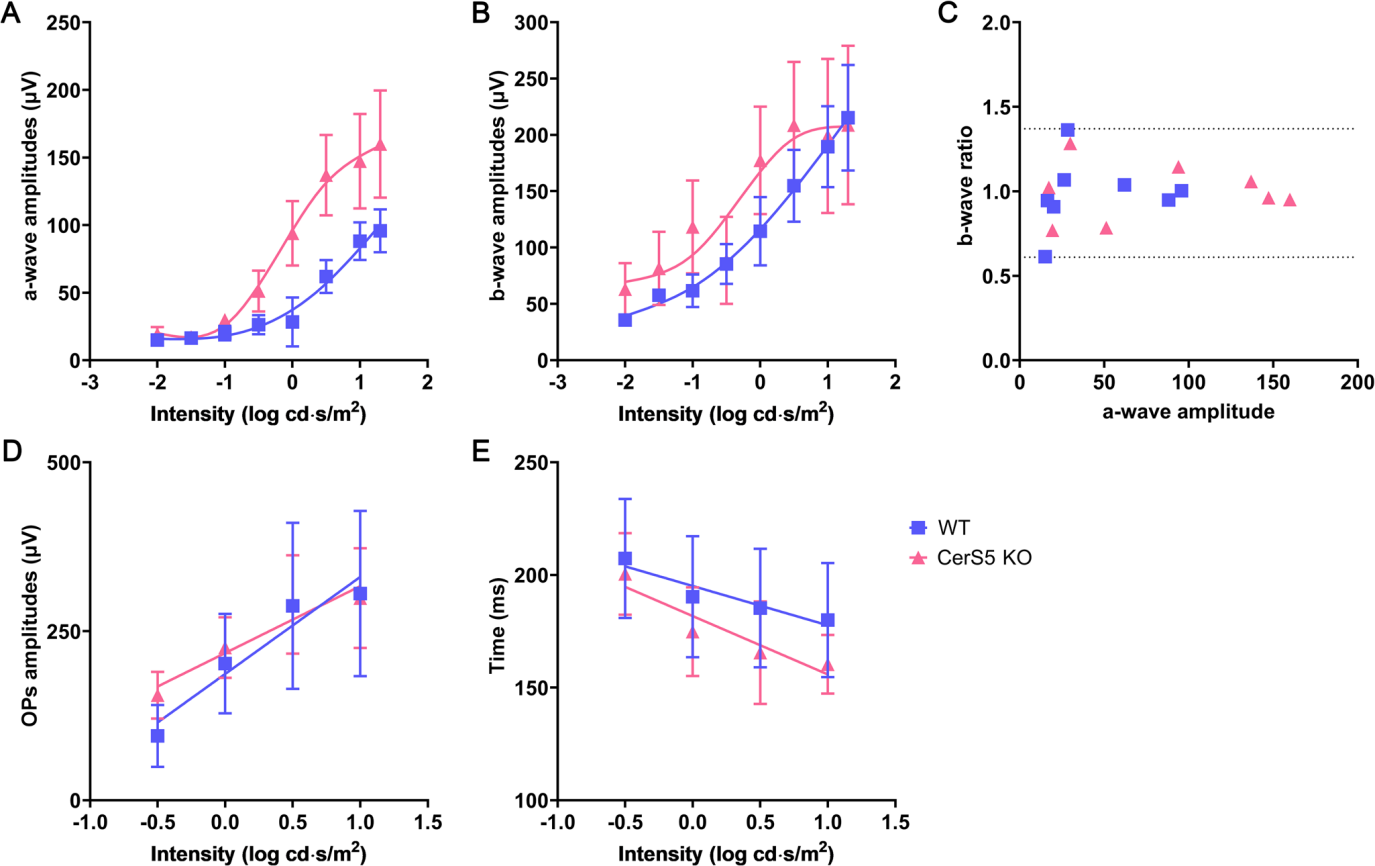
Electroretinographic (ERG) responses from CerS5 wild‐type (WT) and knockout (KO) mouse retinas. Scotopic ERG recordings showing a‐wave (A) and b‐wave (B) amplitudes across flash intensities ranging from −2 to 1.3 log cd s/m^2^. a‐wave amplitudes are presented as absolute values of the negative deflection. CerS5 KO showed a trend toward larger a‐wave and b‐wave amplitudes compared to CerS5 WT retinas. However, these differences did not reach statistical significance (A and B). The b‐wave to a‐wave amplitude ratios were comparable between CerS5 WT and KO retinas and remained within the normal range (dotted line), indicating no abnormal enhancement or reduction in b‐wave relative a‐wave (C). Oscillatory potentials (OPs) were recorded at flash intensities from −0.5 to 1 log cd s/m^2^. OPs amplitude tended to be higher in CerS5 KO retinas than in CerS5 WT retinas (D). Time‐to‐peak of OPs was slightly shorter in CerS5 KO retinas. Neither OPs amplitude nor time‐to‐peak showed statistically significant differences between groups (E).

In scotopic ERG recordings, the amplitudes of both a‐ and b‐waves tended to be higher in CerS5 KO retinas than in WT retinas across the tested flash intensities. The difference in a‐wave amplitudes was most noticeable at −0.5 log cd s/m^2^ and persisted at higher intensities. For the b‐wave, CerS5 KO retinas exhibited larger amplitudes at lower intensities, whereas at 1.3 log cd s/m^2^, the amplitude in KO retinas decreased below that of WT retinas. Despite these trends, no statistically significant differences were observed between the groups at any flash intensity after applying Bonferroni–Dunn correction (Figure 9A,B).

To further investigate potential functional differences, we analyzed the b‐wave ratio, defined as the measured b‐wave amplitude divided by the expected b‐wave value and serves as an indicator of postsynaptic neuronal integrity (Perlman 1983). The b‐wave ratios for both CerS5 WT and KO retinas were distributed within the normal range, with no statistically significant differences between the groups (Figure 9C).

Oscillatory potentials (OPs), which primarily reflect amacrine cell activity within inhibitory feedback pathways mediated by these interneurons (Wachtmeister 1998), were also assessed. OP amplitudes and time‐to‐peak values were measured across flash intensities ranging from −0.5 to 1 log cd s/m^2^ (Hancock and Kraft 2004). OP amplitudes increased with flash intensity in both groups, with CerS5 KO retinas showing a tendency toward higher amplitudes. However, not statistically significant differences were detected between CerS5 WT and KO retinas (*p* > 0.05) (Figure 9D).

Similarly, the time‐to‐peak value decreased as flash intensity increased in both groups, with CerS5 KO retinas exhibiting slightly shorter time‐to‐peak values than CerS5 WT retinas. However, these differences did not reach statistical significance (*p* > 0.05) (Figure 9E).

## 4 Discussion

Although CerS5 has been implicated in various physiological processes, its expression pattern and functional role in the retina remain poorly understood. In the present study, we investigated the expression, localization, and functional significance of CerS5 in mouse retina. X‐gal staining and immunohistochemistry were performed to assess the retinal distribution of CerS5, and ERG was used to evaluate its functional effects on retinal cells.

In CerS5 KO mice, the retina showed a significant reduction in the overall retinal thickness compared with those of WT mice, with decreases observed across all retinal layers except for the OPL. Similar to CerS5 KO mice, Tlcd3b KO mice exhibited retinal degeneration with concomitant reduction of C16‐, C18‐, and C20‐ceramides (Qian et al. 2022, 2023). TLCD3B is a noncanonical CerS and has been reported to be associated with retinal dystrophy. Interestingly, the subretinal reinjection of recombinant adeno‐associated virus eight vectors containing CerS5, but not CerS2 or CerS4, successfully recovered the retinal morphology in Tlcd3b KO mice. Therefore, the retinal thinning observed in CerS5 KO mice is likely attributed to the absence of CerS5, which plays a crucial role in the synthesis and maintenance of C16‐ceramide, as previously reported (Gosejacob et al. 2016). A previous study demonstrated reduced levels of C16:0‐ and C18:1‐ceramide in CerS5 KO retinas than in CerS5 WT retinas (Liu et al. 2024). This depletion of CerS5 may have contributed to the observed retinal thinning, highlighting a potential role for CerS5 in maintaining retinal structural integrity.

Although retinal thinning was evident in CerS5 KO mice, no TUNEL‐positive cells were detected, suggesting that structural changes occurred independently of apoptotic cell death. To further address this discrepancy, soma density was estimated in the ONL, INL, and GCL. Despite layer thinning, soma density was significantly higher in both the ONL and INL of CerS5 KO retinas, indicating that cell number within these layers is likely preserved. These findings suggest that thinning reflects volumetric changes, such as reductions in soma size or extracellular space, rather than cell loss. Interestingly, increased density was also observed in the ONL, where CerS5 is not expressed, implying that the structural effects of CerS5 deficiency may not be entirely cell autonomous. In the GCL, no significant difference in soma density was detected, which may reflect the limited statistical power due to the sparse cell population in this layer. Although these data provide supporting evidence against cell loss, a whole‐mount stereological approach would offer a more definitive assessment of GCL cell number in future studies.

Our study demonstrated that CerS5 is primarily localized in the INL and GCL of the mouse retina, with expression confirmed in the cytoplasm of cholinergic and dopaminergic amacrine cells, horizontal cells, and RGCs. These specific cell types suggest that CerS5 may play an important role in regulating synaptic interactions and neuronal signaling within the retina (Chapot et al. 2017; Kolb 1997). Although highly expressed in certain retinal neurons, the absence of CerS5 does not appear to result in any apparent dysfunction, indicating that CerS5 primarily contributes to maintaining retinal structural integrity rather than directly affecting retinal neuron function.

Selective expression of CerS5 was observed in cholinergic and dopaminergic amacrine cells but was not ubiquitously expressed across all amacrine cell populations. Its selective expression suggests a specialized role in modulating synaptic interactions involving specific interneuron populations, particularly those between bipolar cells, other amacrine cells, and ganglion cells (Kolb 1997). Although OP analysis suggested potential changes in amacrine cell‐mediated signaling (Wachtmeister 1998), these differences were not statistically significant. In addition, although significant structural changes were observed in CerS5 KO retinas, preserved a‐ and b‐wave amplitudes suggest that CerS5 is not functionally involved in PRs or bipolar cells (Bush and Sieving 1994; Stockton and Slaughter 1989), which is consistent with the lack of colocalization in these cell types and further supports the idea that CerS5 KO has minimal effects on rod‐driven signaling in the outer retinal layers (McCulloch et al. 2015). Notably, despite the absence of overt functional impairment, the reduced thickness of the INL and GCL, where CerS5 is expressed, suggests that CerS5 primarily contributes to maintaining retinal structural stability rather than directly influencing neural transmission. Furthermore, the nonsignificant upward trend in ERG amplitudes observed in CerS5 KO retinas may suggest the involvement of compensatory mechanisms, possibly mediated by other CerS isoforms, that help sustain retinal function in the absence of CerS5. Future studies with larger sample sizes will be necessary to determine whether these trends reflect a biologically meaningful compensatory response.

Additionally, we observed a nonsignificant upward trend in the expression of CerS1, CerS2, and CerS4 in CerS5‐deficient retinas. Although these changes did not reach statistical significance, it is possible that they reflect a compensatory response to the loss of CerS5. Future studies with large sample sizes will be necessary to determine the biological significance of these findings.

Of note, although Liu et al. (2024) reported that CerS5 deficiency protected RGCs from damage induced by ocular hypertension, our findings indicate that CerS5 deficiency under conditions is associated with retinal thinning without functional impairment. Moreover, our lipidomic analysis revealed not only a significant reduction in C16‐ceramide levels, as expected, but also a trend toward an increase in C18‐ceramide levels, which contrasts with the findings by Liu et al. who reported a reduction in both C16 and C18:1 ceramides. These differences suggest that the role of CerS5 may be context‐dependent. CerS5 depletion may be beneficial under acute stress conditions, such as elevated intraocular pressure, providing neuroprotection, but in the absence of such stressor, CerS5 deficiency may predispose the retina to structural vulnerability. This context‐specific role underscores the complexity of ceramide metabolism in retinal physiology and highlights the need for further studies to explore how specific ceramide species and environmental stressors interact to influence retinal health.

The structural and cellular findings observed in young adult CerS5 KO mice were further examined at 6 months of age. Total retinal thickness remained significantly reduced in CerS5 KO retinas, with a degree of thinning comparable to that observed in younger mice, suggesting that CerS5 deficiency does not lead to progressive structural deterioration within this time frame. Furthermore, the colocalization between X‐gal signals and specific retinal cell markers was maintained at this later time point, indicating that CerS5 expression patterns remain stable through mid‐adulthood. In this study, retinal thickness measurements were obtained from central retinal sections. Analysis across the full retinal length, including peripheral regions, would be valuable in future studies to more fully characterize the structural effects of CerS5 deficiency. However, as 6 months does not represent an aged stage in mice, and functional analyses were not performed at this time point, it remains to be determined whether CerS5 deficiency contributes to progressive retinal dysfunction over a longer time course. Further studies involving older animals and functional assessments will be necessary to clarify the long‐term consequences of CerS5 loss in the retina.

## 5 Conclusion

This study investigated the distribution and role of CerS5 in the mouse retina and found that CerS5 is predominantly localized in the INL and GCL. Structural changes, including retinal thinning, were observed in CerS5 KO retinas, suggesting that CerS5 is critical for maintaining retinal structural integrity and supporting synaptic stability within the inner retina. Despite these morphological alterations, no significant functional impairments were detected in the absence of CerS5, indicating that compensatory mechanisms may preserve retinal function. Further studies are needed to elucidate the mechanisms underlying the preservation of retinal function in CerS5‐deficient retinas and to assess the potential role of CerS5 in human retinal physiology. A better understanding of the impact of CerS5 on retinal structure and function will provide important insights into its role in retinal diseases.

## Author Contributions

**Soo‐Jin Song**: writing – original draft, investigation, methodology, formal analysis, data curation, validation, visualization, writing – review and editing. **Jae‐Hyun Koo**: methodology, validation. **Hee‐Yeon Kim**: methodology, investigation, validation, visualization. **Joo‐Won Park**: conceptualization, resources, writing – review and editing. **Sun‐Sook Paik**: methodology, investigation, validation, visualization, writing – review and editing. **In‐Beom Kim**: resources, writing – review and editing. **Jung‐A. Shin**: conceptualization, project administration, funding acquisition, supervision, writing – review and editing.

## Conflicts of Interest

The authors declare no conflicts of interest.

## Data Availability

The data that support the findings of this study are available from the corresponding author upon reasonable request.
